# Design of Novel 4-Hydroxy-chromene-2-one Derivatives as Antimicrobial Agents

**DOI:** 10.3390/molecules15064294

**Published:** 2010-06-11

**Authors:** Milan Mladenović, Nenad Vuković, Slobodan Sukdolak, Slavica Solujić

**Affiliations:** Department of Chemistry, Faculty of Science, University of Kragujevac, P.O. Box 60, Serbia; E-Mails: nvukovic@kg.ac.rs (N.V.); duda@kg.ac.rs (S.S.); ssolujic@kg.ac.rs (S.S.)

**Keywords:** 4-hydroxy-coumarins, antimicrobial activity, QSAR, molecular docking, design

## Abstract

This paper presents the design of novel 4-hydroxy-chromene-2 one derivatives, based on previously obtained minimal inhibitory concentration values (MICs), against twenty four microorganism cultures, Gram positive and negative bacteria and fungi. Two of our compounds, **3b** (MIC range 130–500 μg/mL) and **9c** (31.25–62.5 μg/mL), presented high potential antimicrobial activity. The compound **9c** had equal activity to the standard ketoconazole (31.25 μg/mL) against *M. mucedo.* Enlarged resistance of *S. aureus*, *E. coli* and *C. albicans* on the effect of potential drugs and known toxicity of coumarin antibiotics, motivated us to establish SAR and QSAR models of activity against these cultures and correlate biological activity, molecular descriptors and partial charges of functional groups to explain activity and use for the design of new compounds. The QSAR study presents essential relation of antimicrobial activity and dominant substituents, 4-hydroxy, 3-acetyl and thiazole functional groups, also confirmed through molecular docking. The result was ten new designed compounds with much improved predicted inhibition constants and average biological activity.

## 1. Introduction

Coumarins are known potential growth inhibitors of bacteria and fungi, where both natural and synthetic coumarins inhibit growth of common microorganisms *Candida albicans*, *Escherichia coli* and *Staphylococcus aureus* [1,2,3,4,5]. Some coumarin polyacetylene derivatives have antimycobacterial activity against *Mycobacterium fortituum* [6], while others inhibit IQ induced mutation in *S. typhimurium* TA98 [7]. Plant coumarins present antimicrobial potential too [8,9].

The nature of substituents define coumarin activity, and coumarin substitution in positions 3 and 7 is dominant for antimicrobial activity [10]. Our interest is in 3-acetyl and 3-thiazole 4-hydroxy-coumarin derivatives. Beside the known antimicrobial activity [11] of 3-acetyl coumarin derivatives, the presence of thiazole functional group indicates antihelmintic, antibiotic and immunosuppressant activity of a potential drug [12].

While the exact mechanism of coumarin-based microorganism growth inhibition is not yet certain, the 24 kDa N-terminal domain of DNA gyrase B subunit (p24), an enzyme that unwinds double stranded DNA of microorganisms, causing the supercoiling of the DNA, is recognized as the molecular target of natural occurring coumarins like novobiocin and clorobiocin [13] that inhibit the ATPase activity of the B subunit. These drugs have a 4-hydroxycoumarin core in common, which is substituted at the 7 and 3 positions [13]. In p24/novobiocin complex, coumarin ring forms only two hydrogen bonds, both with Arg136 interacting with carbonyl and lactone oxygen [13], while the main interactions occur between p24 and novobiose residue [13]. 

As a result of their varied biological activity, qualitative (SAR) and quantitative structural-activity relationship (QSAR) studies of isolated and synthetic coumarin derivatives are widespread nowadays. Computational studies, which are principals of QSAR, had been performed regarding the molecular lipophilicity [14] and cytotoxic activity [15] of different 4-hydroxycoumarin derivatives. The antimicrobial activity against *S. aureus* and *C. albicans* [16] of some 3-acyl-4-hydroxy and 3-nitrocoumarins has been explained by QSAR, using both semi empirical and density functional theory (DFT) based calculations. Some thiazole coumarin derivatives and their activity against *C. albicans*, *E. coli* and *S. aureus* were the subject of QSAR determination, too [12].

Due to the mentioned activity of carbonyl and thiazole functional groups, we used a synthesized series of carbonyl, carboxyl and thiazole coumarin derivatives (Scheme 1) [3,4] and evaluated their activity through calculated molecular properties and partial atomic charges of potential functional groups, in attempt to explain their bioactivity as antimicrobial agents against Gram positive and negative bacteria and fungus.

Our interest in SAR and QSAR studies of synthetic coumarins started with the examination of the antimicrobial activity of our derivatives [3,4] whereby they presented growth inhibition potential on a wide spectrum of cultures. We decided to perform QSAR evaluation of cultures like *Staphylococcus aureus* (ATCC 25925), *Escherichia coli* (ATCC 25922) and *Candida albicans* (ATCC 10259), for the future design of new more power derivatives. Certain compounds, **3b** [3] and **9c** [4] that displayed both enhanced antibacterial and antifungal activities, can be the base for lead optimization and simple but efficient design of new compounds. 

## 2. Results and Discussion

### 2.1. Antimicrobial activity

**Figure 1:**
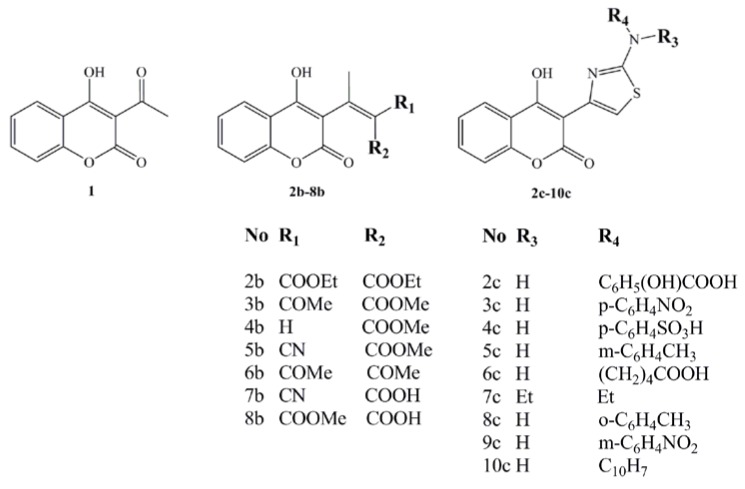
Synthesized 4-hydroxy-coumarin derivatives.

SAR and QSAR methodology were used to explain the antimicrobial activity of the synthesized coumarin derivatives, **1-10c** (Figure 1), on a wide spectrum of the cultures. 

Among the tested compounds, initial coumarin **1** and two structurally different groups of derivatives, first **2-8b**, and second **2-10c**, compounds **3b** and **9c** demonstrated enhanced activity, with MICs in the range of 130–500 and 31.25–62.5 μg/mL, respectively, due to influence of the C-3 coumarin scaffold, acetyl, carboxymetyl and thiazole-m-nitro substituents. The activities of **3b** and **9c** are explained by SAR, QSAR and molecular docking study on DNA gyrase, suggesting them as promising structures for lead optimization and design of new compounds. The carbonyl, carboxyl derivatives **1-8c**, generally, did not presented the expected activity, but their SAR and QSAR studies emphasize the role of coumarin core in activity.

### 2.2. SAR

As presented by Table 1, compounds **2-10c** (MIC range 31.25–500 μg/mL) [4] presented better antimicrobial activity than the compounds **1-8b** (MIC range 90–940 μg/mL) [3]. The main difference in the biological activity of compounds is caused by the presence of thiazole function in **2-10c** followed up with the great dissimilarity in MIC values. The basic compound **1** presented medium activity, with a MIC value of 90 μg/mL for *S. aureus* and *C. albicans*, and 190 μg/mL for *E. coli*. Regarding the overall activity of the first set of synthesized compounds (MIC 130–500 μg/mL), **3b** is the most active one, presenting the same activity due to attendance of carbonyl and carboxyl functions, but lower than **1** against *S. aureus* and *C. albicans* (130 μg/mL), and better against *E. coli* and *M. lysodeikicus* (130 μg/mL). Compared with the reference antibiotics, the activity of **3b** is still much reduced. The insertion of a 2-aminothiazole pharmacophore in the coumarin moiety overcame the low activity of compounds **1-8b** and increased it several times. The most active compound, **9c**, an *m*-nitro derivative, presented an equal MIC value to the reference antibiotic on *M. mucedo* (31.25 μg/mL).

### 2.3. QSAR

The QSAR analysis was performed correlating the antimicrobial activity against *S. aureus* (ATCC 25925), *E. coli* (ATCC 25922) and *C. albicans* (ATCC 10259) presented in Table 1, with various physico-chemical parameters (Table 2) and partial charges (Table 3), to reveal predictions for the lead optimization in the training set of compounds of newly synthesized coumarins [3,4].

**Figure 2:**
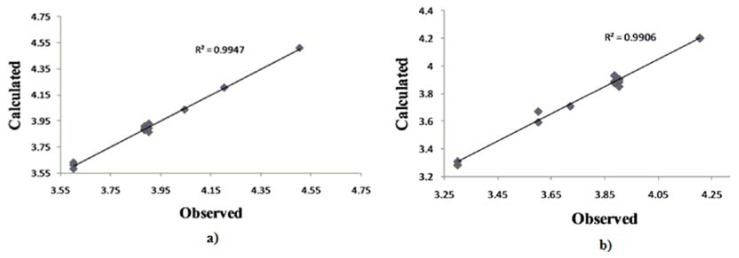
Plot of observed *vs.* calculated – log MIC values of the training set compounds obtained from a) Equation (1), b) Equation (2).

**Figure 3:**
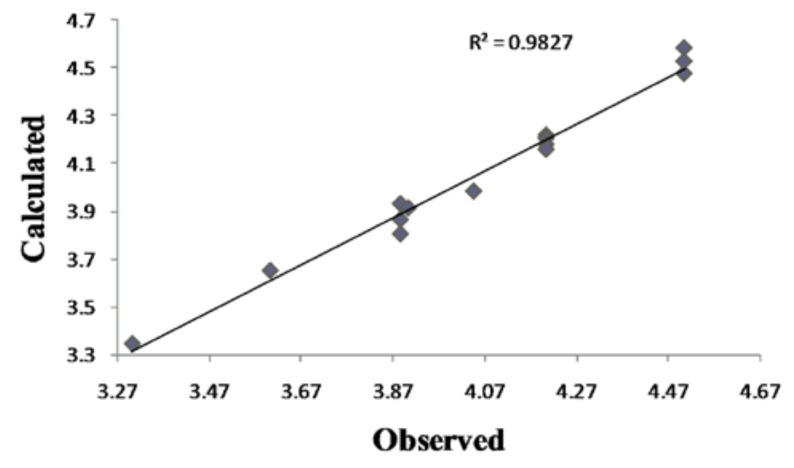
Plot of calculated *vs.* observed – log MIC values of the training set compounds obtained from Equation (3).

Although this set is small, it provides QSAR equations that could be statistically significant for the activity against *S. aureus*, *E. coli* and *C. albicans.* The results of regression analysis are shown in Eqns. (1), (2) and (3) and by Figure 2(a) and (b) and Figure 3, where *n* is number of molecules, *r* is correlation, *F* is Fisher’s significance factor and *s* is standard deviation. Cross-validation resulted with Q^2^ as the square of predictive power of coefficient and s-PRESS as predictive residual sum of squares.

The **model 1** defines the *N*-thiazole atom as important in the interaction of coumarin with bacteria. For the activity against *S. aureus*, the solubility factor is irrelevant. Regarding the **model 2**, besides the *N-*thiazole atom, the antimicrobial activity against *E. coli* depends on interactions with the 4-OH group and *S*-thiazole atom, with solubility and LUMO energy as important factors. The solubility factor is expected referring to Table 2, for it the training set presents wide range in solubility, from the most hydrophilic (-1.765) to the most hydrophobic (3.603) compounds.

The 4-OH and S functional groups do not take the same part in antibacterial activity of **9c** in **model 2**, with correlation factors between biological activity and charge *r*_4-OH_ = 0.603 and *r*_S_= 0.942. The LUMO energy factor suggests that charge transfer might be involved in the mechanism of action [17]. As mentioned, the main factor in the activity is the *N*-thiazole atom, with a correlation *r* = 0.674 of charge and antimicrobial activity of **9c**. Both QSAR equations (1) and (2) for activity against *S. aureus* and *E. coli* are statistically very significant with *F=*104.2987, *r* (99.7%) [Figure 1 (a)] and *F*=28.8394, *r* (99,5%) [Figure 1 (b)], respectively.

QSAR model for activity against *S. aureus* (**model 1**):

- Log MIC = 18.2669( ± 2.82) – 0.0701( ± 0.01) × MR - 0.3670( ± 0.03) + 1.0871( ± 0.30) × HOMO – 0.1682( ± 0.01) × CAA – 0.5095( ± 0.02) × CMA– 7.8116( ± 0.76) × O-lactone + 1.6749( ± 0.44) × N-thiazole – 1.4266( ± 0.23) × S – 0.2159( ± 0.01) × CSEV (1)

n=15 r=0.997 s=0.03 *F*=104.2987 Q^2^=0.995 s-PRESS=0.005

QSAR model for activity against *E. coli* (**model 2**):

- Log MIC = 18.5685( ± 1.95) + 0.9195( ± 0.12) × log *P* – 0.1766( ± 0.04) × lipole - 0.8233( ± 0.15) × virtual log *P* + 1.0722( ± 0.12) × LUMO – 0.0270( ± 0.01) × CAA + 7.0623( ± 1.39) × 4-OH – 8.4340( ± 1.84) × CO-lactone – 1.4566( ± 0.69) × NH + 1.7931( ± 0.81) × N-thiazole – 0.6662( ± 0.47) × S – 22.4376( ± 1.83) × ovality (2)

n=15 r=0.995 s=0.059 *F*=28.8394 Q^2^=0.991 s-PRESS=0.011

QSAR model for activity against *C.albicans* (**model 3**):

- Log MIC = - 3.3032( ± 1.43) + 0.9667( ± 0.10) × log *P* - 1.1485( ± 0.13) - 0.5781( ± 0.10) × LUMO - 0.054( ± 0.01) × CAA + 0.0713( ± 0.01) × CMA - 0.6944( ± 0.24) × 4-OH + 1.2423( ± 0.31) × S + 10.2506( ± 1.41) × ovality (3)

n=15 r=0.991 s=0.068 *F*=42.6548 Q^2^=0.983 s-PRESS=0.028

Antifungal activity against *C. albicans,* equation Eqn. (3), can be explained by influence of solubility [18], electronic (LUMO) and steric (CMA) parameters. The positive impact of the *S*-thiazole atom on QSAR **model 3** confirms the thiazole group as the most important one in the coumarin derivatives’ activity. The lower (**model 3)** or even no **(model 1**) influence of 4-OH might indicate a different coumarins-fungus mechanism of interaction compared to the coumarin-bacteria one (**model 2**). The goodness-of-fit of equation (3) (Figure 2) is very significant, possessing a high *r* (99.1%) and a small *s* (0.068) with an overall *F* test value of 42.6548 at the significant level of *p* < 0.05. The correlation matrix of biological activity with molecular descriptors is presented in Table 4.

The linear regression analysis of the activity of the most active compound, **9c**, presents the correlation of *m*-nitro group and biological activity with the value of *r* = 0.902. This value is the highest correlation coefficient for partial charge found in this study and it overlaps with the microbiology and SAR results. The QSAR results are in high correlation with SAR and they confirmed the SAR statement that the thiazole ring is the key part for antimicrobial activity. The absence of carbonyl and carboxyl functions (compounds **1-8b**) in the QSAR equations verified them as particularly unimportant for antimicrobial activity.

### 2.4. Molecular docking

Crystallographic data of the binding of novobiocin and chlorobiocin show four hydrogen bonds between novobiose and p24 (Asn46, Gly50, Asp73), but only two between the enzyme and coumarin ring formed by lactone part and Arg136 [13]. We performed the molecular docking of **1**, **3b** and **9c** to obtain interactions of our derivatives in active site of 24 kDa N-terminal domain of DNA gyrase B subunit and to explain the activity of the compounds, aiming interactions of C-3 scaffolds with Arg136 when grid box was set to cover the crucial amino acids for cumarin binding, *i.e.*, Arg76, Pro79 and Arg136 [13]. 

**Figure 4:**
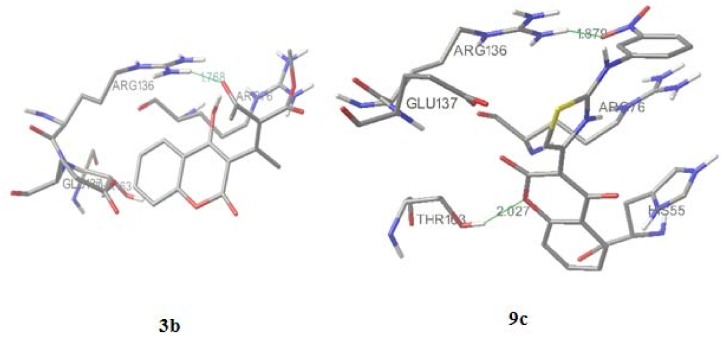
Molecular docking of compounds **3b** and **9c.**

Compound **1** forms with Arg136 only one hydrogen bond, between the lactone O atom of the coumarin ring and the NH_2_ guanido atom of Arg136. The activity of **3b** (Figure 4) is expressed also with formation of one hydrogen (1.768 Å) bond with Arg136, formed by the carbonyl oxygen of the scaffold. Also, the guanido carbon forms with the C-5 coumarin carbon a pi-cation interaction. Predicted inhibition constant for **3b**, *K_i_*= 1.5 mM *versus* the inhibtion constant of **1**, *Ki* = 3.67 mM, and the role of residue confirm SAR and QSAR interpretations of the highter activity of **3b** related to **1**.

The most active compound, **9c** (Figure 3), forms two hydrogen bonds, making the great difference in overall activity. The first one (2.027 Å) is between the lactone O and anew amino acid, Thr163, opposite to **1** and **3b**. The second one (1.879 Å) is formed by the *m*-NO_2_ oxigen atom and Arg136, which is the final conformation of the high influence of thiazole-*m*-nitro system on activity of **9c**, also presented by SAR and QSAR. The sulfur from the thiazolee ring forms ionic interactions with Gly 137. The orientations of Glu50, Arg76 and Pro79 around **1**, **3b** and **9c** are similar to the ones around novobiocin, mostly forming hydrophobic interactions [13].

### 2.5. Design of novel coumarin derivatives

Based on obtained SAR, QSAR and molecular docking results, we designed ten new compounds with high improved predicted *Ki* values and average antimicrobial activity. The design of new compounds, with the aim to remove the novobiose residue from antimicrobial activity of the coumarins and passing it on the C-3 scaffolds, had been performed respecting simple principles: introduction of hydroxyl group in C-7 position of the coumarin core, replacement of inactive **b** compounds 3-(prop-1-en-2-yl) function by nitrogen or sulfur more active residues, activation if inactive of carbonyl and carboxyl residues and retention or further modification of active pharmacophores learned by SAR and computational studies.

**Figure 6:**
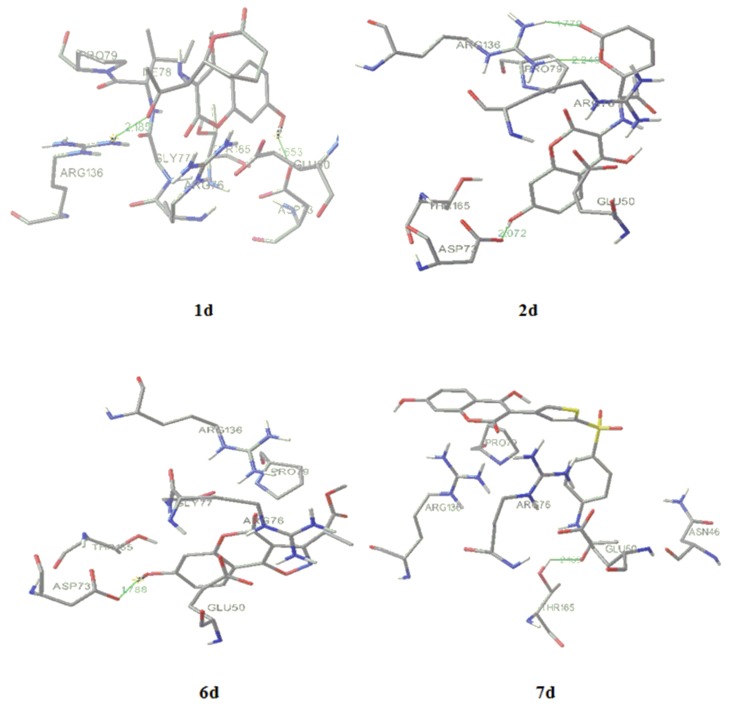
Molecular docking of the most active designed compounds **1d**, **2d**, **5d** and**7d**.

All the structures were optimized and docked according to described methods (Figure 6). The range of predicted average MIC was 3–25 μg/mL, far below the activity obtained by experiments. The most active designed compound (Figure 6), **7d**, with predicted *Ki* = 90.06 µM and MIC = 3 μg/mL forms with p24 one hydrogen bond (green line on Figure 6) between the *p*-N-acetyl oxygen and Thr165 (2.169 Å). Following compound, **1d**, *Ki* = 7.87 µM and MIC = 10 μg/mL, forms two hydrogen bonds with enzyme, between the carbonyl residue and Arg136 (2.185Å) and between 7-OH and Asp73 (1.653 Å). The increased number of hydrogen bonds formed by **2d**, *i.e.*, one 7-OH-Asp73 (2.072 Å) interaction and two interactions between Asp136 and external lactone ring oxygen atoms (1.779; 2.249 Å), could improve drug activity (*Ki* = 23.06 µM and MIC = 15 μg/mL). The formation of hydrogen bonds between introduced 7-hydroxyl group (**5d**; *Ki* = 96 µM and MIC = 25 μg/mL and **6d**; *Ki* = 128 µM and MIC = 18 μg/mL, 1.788Å) and Asp73 and electrostatic interaction between the oxazole ring and Arg136 (**6d**) enlarge the antimicrobial potential, while only bonding 7-OH-Arg136 cannot explain high activity at compound **10d**. The activity of **3d (***Ki* = 428.36 µM and MIC = 25 μg/mL) would depend of hydrogen bind ability of coumarin core.

## 3. Experimental

### 3.1. Synthesis

In our previous papers, synthesis and pharmacological evaluation of the group of 16 substitued potential anticoagulant chromene-*2H*-one derivatives **1-10c** were reported (Scheme 1) [3,4], underlining that two different synthetic approaches used, microwave assisted Knoevenagel condensation (compounds **1-8b**) [3] and Hantzsch reaction (compounds **2-10c**) [4]. Both methods are efficient in functionalisation of C-3 position in the coumarin moiety. Compounds were characterized by elemental analyses (C, H, N, O and S) with determination of molecular weights by mass spectroscopy (MS). Structural characterisation was performed by infrared spectroscopy (IR), ^1^H- Nuclear Magnetic Resonance (NMR) and MS spectra. 

### 3.2. Assay for in vitro antimicrobial activity

The antimicrobial activity of synthesized coumarins was measured using the microdilution two-fold method in Mueller-Hinton broth for bacteria and Saboruaud dextrose broth for fungi [19,20] at pH = 7.4. Antimicrobial targets were both Gram positive and negative bacteria and fungi, using wide range of ATCC cultures and clinical isolates [3,4], using Resazurin solution (0.02 mL of 0.05% concentration) [21] for determination of MICs (Table 1).

### 3.3. Molecular modeling

A data set of 15 compounds has been taken from published articles [3,4]. Preliminary molecular descriptor characterisation of compounds **1-8b** has been performed [3] by the PM3 semiempirical method, but those values were not used in this paper. The initial structures were built in Spartan 2002 for Windows [22] then imported in MacroModel [23] to adjust and minimize atom and bond types with the OPLS 2005 force field parameters. The generalized Born/surface area (GB/SA) continuum non solvent model was used to simulate vacuum environment, consequently due to insolubility of the tested compounds in water, but to provide proper optimization according to experiment conditions [3,4]. An extended non-bonded cutoff (van der Waals 8 Å; electrostatic 20 Å) was used. Systematic conformational search on each molecule was performed by 500 step Monte Carlo conformational analysis, 100 steps per rotatable bond, with the energy cutoff generally set to Δ*E* = 10 kJ/mol above the lowest energy conformation. 

Further, the geometry optimization was performed by MOPAC PM6 [24] Hamiltonian semi empirical method imported in Vega ZZ [25], by fixing gradient norm as 0.01, with ε = 24.3 and ε = 48.0, in order to include solubility of compounds **1-8b** in ethanol [3], and **2-10c** in DMSO [4]. The most stable structures, with determined final heat of formation, were selected as representative conformations in calculation of molecular descriptors in this study.

The CS Gaussian 03 program [26] by density functional theory (DFT) using the B3LYP hybrid functional including 6-31G (d) basis with Mulliken population analysis was applied for partial atomic charge computations of the optimized models. 

The virtual log *P*, Broto log *P*, lipole, molar refractivity, dipole and ovality of all single molecules were evaluated by VegaZZ software. Steric descriptor values like Connolly Accessible Area (CAA), Connolly Molecular Area (CMA) and Connolly Solvent-Excluded Volume (CSEV) [12,27], were calculated with Chem3D Ultra 10.0/ ChemOffice 2006 software package [28].

Single and multivariable linear regression fits were estimated between –Log MIC values and molecular descriptors and calculated partial charges. Good correlation between biological activity and partial charge indicates functional group as the active one [29]. Origin Pro 8 software [30] used to generate QSAR models by MRA. Cross validation was performed using leave-one-out method.

### 3.4. Molecular docking

The crystal structure of *E.coli* 24 kDa N-terminal domain of DNA gyrase B subunit in complex with clorobiocin from the Brookhaven Protein Data Bank (PDB ID code: 1KZN) was used in docking experiments [31]. Crystallographic waters and inhibitor which were not hydrogen-bonded to the enzyme were removed with Chimera software [32]. Initially, compounds **1**, **3b** and **9b** were used as the ligands for docking; following by docking of the newly designed and optimized structures **1-10d**. The docking was performed with version 4.0 of AutoDock [33], which combines a rapid energy evaluation through precalculated grids of affinity potentials with a variety of search algorithms to find suitable binding positions for a ligand on given protein. The dimensions of the grids were thus 34 Å x 34 Å x 34 Å, with a spacing of 0.375 Å between the grid points and center grid box coordinates x = 17.507, y = 32.5, z = 38.164. Lamarckian Genetic Algorithm was use for docking, with number of GA runs set to 100 and RMS Cluster Tolerance of 0.5 Å.

## 4. Conclusions

Based on previously obtained antimicrobial activity of fifteen 4-hydroxycoumarin derivatives, we performed the SAR, QSAR studies on their activity against *S. aureus*, *E. coli* and *C. albicans*, and molecular docking studies on the antimicrobial activity molecular target. The studies emphasize the important roles of the C-3 carbonyl and thiazole residues in activity, which led to the design of ten new potentially active coumarin antimicrobial agents. The designed compounds have improved predicted *Ki* values and antimicrobial activity, which will be confirmed through further synthesis and determination of real antimicrobial activity.

## Figures and Tables

**Figure 5:**
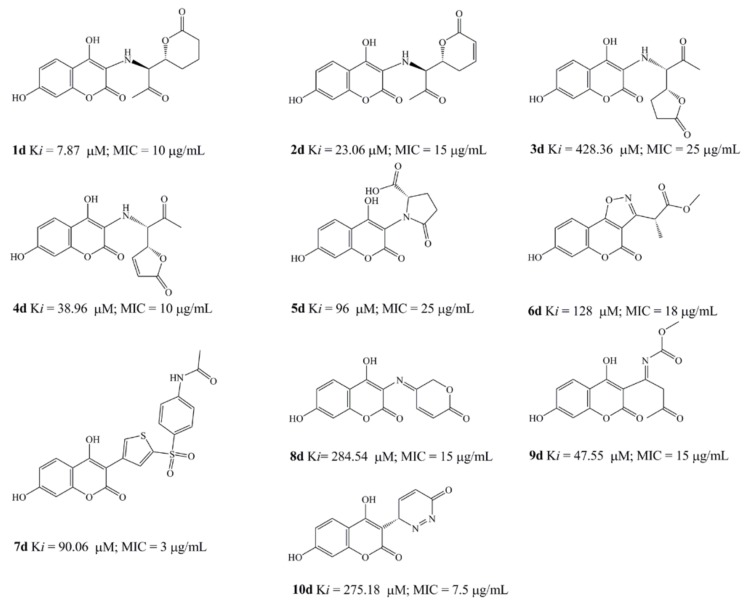
Designed 4-hydroxycoumarin derivatives.

**Table 1:** Antimicrobial activity of synthetic coumarin derivatives.

	*S. aureus*			*E. coli*			*C. albicans*	
Comp.		MIC^a^ values of tested compounds (10^-6^ g/mL) (-log MIC)						
	Experimental	Calculated	Experimental		Calculated	Experimental		Calculated
**1**	90 ± 0.35 (4.046)	103 (3.984)	190 ± 0.35 (3.721)		195 (3.708)	90 ± 0.05 (4.046)		92 (4.035)
**3b**	130 ± 0.22 (3.886)	156 (3.806)	130 ± 0.34 (3.886)		132 (3.878)	130 ± 0.15 (3.886)		130 (3.885)
**4b**	130 ± 0.50 (3.886)	222 (3.652)	130 ± 0.25 (3.886)		129 (3.888)	250 ± 0.25 (3.602)		132 (3.878)
**6b**	130 ± 0.45(3.886)	116 (3.932)	250 ± 0.55(3.602)		255 (3.592)	130 ± 0.15 (3.886)		129 (3.889)
**7b**	130 ± 0.35 (3.886)	112 (3.947)	500 ± 0.35 (3.301)		519 (3.284)	500 ± 0.35 (3.301)		124 (3.908)
**8b**	130 ± 0.26 (3.886)	136 (3.865)	130 ± 0.35 (3.886)		117 (3.932)	130 ± 0.55 (3.886)		130 (3.886)
**2c**	125 ± 0.25 (3.904)	62.5 (4.206)	250 ± 0.55 (3.602)		491 (3.309)	62.5 ± 0.55 (4.204)		137 (3.862)
**3c**	125 ± 0.25 (3.904)	60 (4.217)	62.5 ± 0.25 (4.204)		62.5 (4.204)	62.5 ± 0.55 (4.204)		145 (3.893)
**4c**	62.5 ± 0.29 (4.204)	62.5 (4.204)	62.5 ± 0.10 (4.204)		62.6 (4.203)	62.5 ± 0.15 (4.204)		63 (4.205)
**5c**	62.5 ± 0.24 (4.204)	33.5 (4.475)	125 ± 0.50 (3.904)		124 (3.906)	31.25 ± 0.10 (4.505)		63 (4.205)
**6c**	250 ± 0.35 (3.602)	66 (4.179)	125 ± 0.35 (3.904)		125 (3.902)	62.5 ± 0.15 (4.204)		234 (3.630)
**7c**	250 ± 0.55 (3.602)	26.2 (4.581)	125 ± 0.45 (3.904)		131 (3.884)	31.25 ± 0.55 (4.505)		245 (3.610)
**8c**	250 ± 0.36 (3.602)	121 (3.914)	250 ± 0.12 (3.602)		214 (3.670)	125 ± 0.15 (3.904)		262 (3.582)
**9c**	31.25 ± 0.21 (4.505)	30 (4.526)	62.5 ± 0.09 (4.204)		63.1 (4.200)	31.25 ± 0.25 (4.505)		31 (4.508)
**10c**	125 ± 0.38 (3.903)	69.5 (4.158)	125 ± 0.25 (3.904)		141 (3.851)	62.5 ± 0.55 (4.204)		118 (3.928)
**S**	31.25 ± 0.07		31.25 ± 0.35					
**K**						1.95 ± 0.05		

^a^ Results are mean values SD from at least three experiments; ^b^S=streptomycin; ^c^K=ketoconazole.

**Table 2:** Relevant calculated physico-chemical parameters of synthesized 4-hydroxy-coumarin derivatives.

Comp.	log *P*	MR	lipole	HOMO	LUMO	CAA	CMA	CSEV	ovality
**1**	-0.529	51.062	2.161	-9.989	-1.490	335.170	156.104	124.411	1.368
**3b**	-1.318	75.494	1.622	-10.015	-1.562	466.977	236.476	203.327	1.590
**4b**	-0.035	66.062	1.646	-9.958	-1.537	424.305	205.095	165.182	1.495
**6b**	-1.679	75.277	1.905	-9.977	-1.453	444.049	223.344	192.322	1.561
**7b**	-1.765	67.538	2.645	-10.040	-1.646	424.466	207.570	171.656	1.482
**8b**	-1.709	66.570	2.507	-10.037	-1.694	426.269	208.634	173.406	1.512
**2c**	1.216	96.238	0.557	-9.042	-1.449	566.431	291.664	246.339	1.645
**3c**	3.129	95.320	2.244	-9.003	-1.954	542.789	279.873	233.719	1.604
**4c**	0.702	65.468	1.415	-8.999	-1.621	572.035	296.286	249.537	1.654
**5c**	2.904	95.722	2.513	-8.900	-1.135	521.823	269.577	225.940	1.608
**6c**	1.921	88.488	0.281	-8.923	-0.936	552.855	279.213	230.128	1.680
**7c**	1.856	84.807	1.112	-8.890	-0.950	478.322	244.811	208.372	1.588
**8c**	3.380	93.722	2.552	-8.913	-1.072	521.339	268.518	225.738	1.604
**9c**	3.129	95.320	2.101	-8.972	-1.759	538.849	281.111	237.198	1.614
**10c**	3.603	104.966	3.169	-8.866	-1.231	558.547	292.497	247.999	1.641

**Table 3:** Partial atomic charges of the compounds **1-8b** and **2-10c**.

	Partial atomic charges of the compounds								
**Functional groups**	**1**	**3b**	**4b**	**6b**	**7b**	**8b**			
4-OH	-0.615	-0.646	-0.637	-0.648	-0.614	-0.625			
O-lactone	-0.523	-0.523	-0.522	-0.522	-0.515	-0.515			
CO-lactone	-0.474	-0.491	-0.493	-0.491	-0.473	-0.464			
CO	-0.456	-0.609	-0.604	-0.613		-0.531			
		0.221	0.235	0.225					
CO-carboxyl		-0.743	-0.747	-0.745	-0.456	-0.488			
OH-carboxyl		-0.507	-0.492		-0.579	-0.586			
CN		-0.452	-0.463		-0.475				
**Functional groups**	**2c**	**3c**	**4c**	**5c**	**6c**	**7c**	**8c**	**9c**	**10c**
4-OH	-0.638	-0.644	-0.204	-0.617	-0.675	-0.671	-0.646	-0.637	-0.648
O-lactone	-0.522	-0.520	-0.114	-0.599	-0.531	-0.532	-0.523	-0.522	-0.522
CO-lactone	-0.492	-0.490	-0.195	-0.499	-0.515	-0.516	-0.491	-0.493	-0.491
N-thiazole	-0.582	-0.604	-0.261	-0.681	-0.673	-0.659	-0.609	-0.604	-0.613
S-thiazole	0.230	0.244	0.296	0.458	0.261	0.259	0.221	0.235	0.225
N-amine	-0.750	-0.744	-0.262	-0.753	-0.610	-0.419	-0.743	-0.747	-0.745
CO-carboxyl	-0.467				-0.468				
OH-carboxyl	-0.582				-0.563				
OH-phenyl	-0.638								
N-nitro		-0.374						-0.386	
O-nitro		-0.400^b^						-0.392^b^	
		-0.401^6^						-0.386^b^	
S-SO_3_H			0.758						
O-SO_3_H			-0.282^c^						
			0.259^c^						
OH-SO_3_			-0.256						

^a^Two carbonyl groups in compound **6b**; ^b^Two O-nitro atoms in compounds **3c** and **10c**; ^c^Two O-SO_3_H atoms in compound **4c.**

**Table 4:** Correlation matrix of biological activity with molecular descriptors.

	^a^D1	D2	D3	D4	D5	D6	D7	D8	D9	D10	D11	D12	D13	D14	^b^C1	C2	C3
**D1**	1.00																
**D2**	0.43	1.00															
**D3**	0.35	0.99	1.00														
**D4**	0.39	0.35	0.25	1.00													
**D5**	0.77	0.54	0.46	0.45	1.00												
**D6**	0.64	0.59	0.43	0.46	0.98	1.00											
**D7**	0.35	0.43	0.47	0.44	0.96	0.96	1.00										
**D8**	0.56	0.37	0.11	0.78	0.37	0.19	0.23	1.00									
**D9**	0.12	06	0.66	0.12	0.54	0.43	0.46	0	1.00								
**D10**	0.28	0.95	0.89	0.23	0.62	0.61	0.22	0	0.94	1.00							
**D11**	0.11	0.72	0.68	0.27	0.63	0.64	0.58	0	0.36	0.44	1.00						
**D12**	0.29	0.47	0.34	0.61	0.15	0.15	0.19	0	0.18	0.19	0.36	1.00					
**D13**	0.36	0.44	0.95	0.25	0.31	0.37	0.31	0	0.62	0.55	0.34	0.95	1.00				
**D14**	0.13	0.78	0.66	0.14	0.72	0.74	0.64	0.15	0.73	0.78	0.66	0.23	0.47	1.00			
**C1**	045	0.52	0.94	0	0.44	0.53	0.57	0	0	0.84	0.32	0.36	0.96	0.66	1.00		
**C2**	0.77	0	0	0.88	0.94	0.51	0.53	0.96	0.54	0.26	0.58	0.21	0.19	0.34	0.55	1.00	
**C3**	0.75	0	0	0.96	0.12	0.35	0.15	0.93	0.24	0	0	0	0	0.1	0.52	0.75	1.00

^a^D1 log *P*; D2 MR; D3 HOMO; D4 LUMO; D5 CAA; D6 CMA; D7 CSEV; D8 ovality; D9 4-OH; D10 O-lactone; D11 CO-lactone; D12 NH; D13 N-thiazole; D14 S; ^b^C1 *S. aureus*; C2 *E. coli*; C3 *C. Albicans.*
